# Organic Fertilization and Sufficient Nutrient Status in Prehistoric Agriculture? – Indications from Multi-Proxy Analyses of Archaeological Topsoil Relicts

**DOI:** 10.1371/journal.pone.0106244

**Published:** 2014-09-02

**Authors:** Franziska Lauer, Katharina Prost, Renate Gerlach, Stefan Pätzold, Mareike Wolf, Sarah Urmersbach, Eva Lehndorff, Eileen Eckmeier, Wulf Amelung

**Affiliations:** 1 Institute of Crop Science and Resource Conservation – Soil Science and Soil Ecology, University of Bonn, Bonn, Germany; 2 Archaeological Heritage Management Rhineland (LVR-Amt für Bodendenkmalpflege im Rheinland), Bonn, Germany; 3 Department of Geography, RWTH Aachen University, Aachen, Germany; Institute of Tibetan Plateau Research, China

## Abstract

Neolithic and Bronze Age topsoil relicts revealed enhanced extractable phosphorus (P) and plant available inorganic P fractions, thus raising the question whether there was targeted soil amelioration in prehistoric times. This study aimed (i) at assessing the overall nutrient status and the soil organic matter content of these arable topsoil relicts, and (ii) at tracing ancient soil fertilizing practices by respective stable isotope and biomarker analyses. Prehistoric arable topsoils were preserved in archaeological pit fillings, whereas adjacent subsoils served as controls. One Early Weichselian humic zone represented the soil status before the introduction of agriculture. Recent topsoils served as an additional reference. The applied multi-proxy approach comprised total P and micronutrient contents, stable N isotope ratios, amino acid, steroid, and black carbon analyses as well as soil color measurements. Total contents of P and selected micronutrients (I, Cu, Mn, Mo, Se, Zn) of the arable soil relicts were above the limits for which nutrient deficiencies could be assumed. All pit fillings exhibited elevated δ^15^N values close to those of recent topsoils (δ^15^N>6 to 7‰), giving first hints for prehistoric organic N-input. Ancient legume cultivation as a potential source for N input could not be verified by means of amino acid analysis. In contrast, bile acids as markers for faecal input exhibited larger concentrations in the pit fillings compared with the reference and control soils indicating faeces (i.e. manure) input to Neolithic arable topsoils. Also black carbon contents were elevated, amounting up to 38% of soil organic carbon, therewith explaining the dark soil color in the pit fillings and pointing to inputs of burned biomass. The combination of different geochemical analyses revealed a sufficient nutrient status of prehistoric arable soils, as well as signs of amelioration (inputs of organic material like charcoal and faeces-containing manure).

## Introduction

Human activities had an impact on landscapes since the Neolithic, e.g. by the construction of settlements, arable cropping and animal husbandry. As a result, also the chemical composition of the soils changed [1]–[3]. These chemical changes of ancient soils may still be preserved in buried soils and, therefore, may be used to elucidate human activities inside and outside prehistoric settlements, for instance by analyzing prehistoric topsoil relicts. Buried ancient topsoil relicts can be preserved as fillings of pits that have been constructed outside prehistoric settlements. These pits are defined as off-site features that do not contain any characteristic anthropogenic artefacts, settlement material, or settlement related nutrient inputs. They have been part of the prehistoric agrarian landscape and therefore their dark humic soil filling presumably consists to a large extent of prehistoric topsoil from an open landscape that was most likely arable land [4]–[6]. Some of these arable topsoil relicts were buried in deep pit features, and hence have largely been preserved from the influence of roots, bioturbation and weathering processes by overlying sediments [6]. Examples for deep off-site features are man-made slot pits (“Schlitzgruben”) and man-made pit alignments located outside prehistoric settlements [4], [7] in which relocated topsoil material that bears information on prehistoric nutrient status and fertilization measures has been conserved as the infilling of those pits [8]. It was shown that pit fillings investigated in German archaeological excavation sites usually exhibited larger contents of soil organic carbon, total nitrogen, as well as extractable and organic phosphorus compared with the adjacent subsoils [4], [8]. However, the origin of this organic matter remained unclear.

Geochemical analyses of ancient topsoils have been conducted since the 1930s as means of archaeological prospection – mainly in settlement areas [9]–[11]. In recent years new methods have enlarged the spectrum of analyses for archaeological soil material. The mapping of activity areas within settlements on the basis of soil phosphorus (P) concentrations is an established method in archaeology (for general overviews see [12]–[15]). However, the differentiation between the various P fractions has scarcely been explored so far [16], especially in off-site features like slot pits and pit alignments [8]. Elevated extractable P (i.e. sum of all sequentially extracted inorganic and organic P fractions) and plant available inorganic P contents (i.e. sum of resin-P_i_, NaHCO_3_-P_i_ and NaOH-P_i_) in ancient topsoil relicts compared with control samples can be seen as first hints for soil amelioration in prehistoric times [8]. Yet, it remained uncertain to which degree elevated contents of organic P were the result of a P-cycling from mineral phases by plants or microorganisms, or an indication of organic fertilization. For the differentiation between both sources, w more information on other nutrients that are less abundant in the organic phase (like micronutrients) and on the origin of soil organic matter (SOM) preserved within prehistoric topsoils are required.

Several geochemical methods for characterizing soil micronutrient contents and for detecting traces of fertilization are nowadays available. Therefore, we combined the analyses of different chemical markers in archaeological soil samples. Some micronutrients possess a low mobility in soils, and are therefore suitable to track past human activities in settlement areas, e.g. to separate areas of food preparation, hearths, manuring or craft working [17], [18]. Depending on the soil properties in distinct regions, even human health can be affected by micronutrient deficiency [19], e.g. the iodine content of food depends on the iodine content of the soil in which it is grown. Prehistoric fertilization using nitrogen fixing plants might be evidenced by measurements of stable nitrogen isotope and the amino acid composition in ancient topsoil material. This is possible because legumes fix relatively more of the “light” ^14^N isotope, leading to a changed ^14^N to ^15^N ratio [20]–[22], and the amino acid composition may be specific for distinct crops like, e.g. legumes [23]–[25]. Moreover, compound-specific δ^15^N analysis of amino acids in soil has been used to identify land use and manure application in archaeological contexts (i.e. in Medieval and Bronze Age time periods) [26]. Beside the cultivation of legumes, application of livestock manure or human faeces might have been practiced to preserve and enhance soil fertility in prehistory. Measurements of specific steroids, i.e. 5β-stanols and bile acids, provide a reliable tool to identify ancient faecal deposition. Among the steroids, 5β-stanols such as coprostanol and 5β-stigmastanol were found to be indicators for the faecal input of omnivores and herbivores species, respectively [27]–[30]. However, there are studies that could also show a contribution of microbial sterol degradation to an enhanced 5β-stanol signal in sediments and sewage sludge [31], [32]. Therefore, there is most likely a natural background concentration of 5β-stanols in soils. Similarly to 5β-stanols, stanones are also thought to be indicative for the input of faeces because they are generated both via intestinal and sedimentary processes of sterol reduction [33]–[35]. Additionally, bile acids serve as faecal markers because they are exclusively formed by vertebrates, are presumed to exhibit an greater resistance to degradation than 5β-stanols [36], and even allow to distinguish between different faecal sources (human, ruminant, and porcine) [28]–[30], [37]. Up to now there are only few studies that have used stanones as biomarker [35], [38], [39]. Even fewer studies investigated bile acids to determine manuring in prehistory, because of the requirement of a complex chemical isolation procedure [40].

The burning of the fields was another activity connected to prehistoric farming, as it allowed to clean the soils from native vegetation, to fertilize them and to fight weeds [41], [42]. These former slash-and burn practices can be evidenced by the combination of black carbon analysis and soil color measurements [7], [43]–[45]. The objective of this study was to contribute to a better understanding of the nutrient status and organic matter properties of ancient topsoil relicts in archaeological pit fillings using a combination of different geochemical analyses. We hypothesized that prehistoric agriculture left fingerprints of altered soil nutrient contents and fertilization. Therefore, we (i) elucidated the total P and micronutrient status of prehistoric arable soils, and ii) characterized the origin of soil organic matter using natural δ^15^N abundance as markers for legume cultivation and manure application [46], [47], amino acid composition as markers for the cultivation of legumes [23]–[25], benzene polycarboxylic acids and color measurements as markers for charred remains in soils [7], [43]–[45], and sterols and bile acids as markers for faecal residues [1], [30], [48], [49].

## Materials and Methods

For the described study all necessary permits were obtained from the Museum and Archaeological State Service of Saxony-Anhalt and the Archaeological Heritage Management Rhineland, which complied with all relevant regulations.

### 1 Sites and archaeological topsoil relicts

Selected archaeological topsoil relicts (i.e. fillings of slot pits and pit alignments) were investigated from two prehistoric settlement areas in Germany: Central (now comprising Chernozems and Phaeozems) and Western Germany (now comprising Luvisols) (Table 1; see also Lauer *et al.* [8]). Both regions had been settled since Early Neolithic time by sedentary farmers. The studied soil relicts were preserved in deep features such as slot pits found in the surroundings of Middle and Younger Neolithic sites (i.e. around 7,000 until 5,500 before present (BP); [50], [51]) and pit alignments dating to the Younger Bronze Age or Early Iron Age (between 3,200 and 2,700 BP; [52]). Both slot pits and pit alignments are enigmatic features, situated outside prehistoric settlements presumably in the areas of prehistoric arable fields (off-site features) and normally do not contain any archaeological artefacts. Until now, little is known about the function of the slot pits [7], [50], [53]. They have a slot-like appearance, being up to 3 m deep, 2 m long and narrow (0.2–1 m), and they are defined as anthropogenic due to their regular and specific shape and because they often appear in regularly arranged groups [50], [51]. With respect to their narrow shape it is presumed that they remained open for only a few days to several weeks and were refilled with autochthonous topsoil and subsoil materials, either on purpose, via erosion, or both. Humic soil material ( = ancient topsoil material) in archaeological features like pits or ditches is regarded as prominent archaeological finding [4]–[6], [54].

**Table 1:** Characterization of the study sites.

Region/ excavation site	Number of pits	Type of soil archive	Age of topsoil relicts	Geographical coordinates	MAP^a^ mm	MAT^b^°C	Soil parent material
**Central Germany**							
***Chernozem region***							
Kleingräfendorf	2	Pit alignment	Bronze Age	51° 22′ N, 11° 51′ E	<500^c^	8.5–9^c^	loess
Oechlitz	1	Pit alignment	Bronze Age	51° 19′ N, 11° 45′ E			loess
Jüdendorf	3	Pit alignment	Bronze Age	51° 18′ N, 11° 42′ E			loess
***Phaeozem region***							
Prießnitz	2	Slot pit	Neolithic	51° 6′ N, 11° 46′ E	560–580^c^	8–8.5^c^	loess
**Western Germany**							
***Luvisol region***							
Merzenich^e^	6	Slot pit	Neolithic	50° 49′ N, 6° 32′ E	650–700^d^	10–11^d^	loess
Pulheim	2	Slot pit	Neolithic	50° 59′ N, 6° 48′ E			loess
Düren Arnoldsweiler (humic zone)^e^	1	Humic zone	Early Weichselian	50° 51′ N, 6° 30′ E			loess

a MAP = mean annual precipitation;

b MAT = mean annual temperature;

c Kropp *et al.* [133];

d Genßler *et al.* [134],

e excavation sites were selected for steroid analyses.

The Bronze Age pit alignments are evenly spaced pits arranged in lines which run for large distances in straight or curving lines [52]. Again, little is known about their original function [52]. Like in the case of the slot pits, also the pit alignments are clearly anthropogenic as proved by their shape [52]. Similarly to the slot pits the humic filling of these pits represents the prehistoric topsoil in off-site positions [50], [51]. More detailed information about the archaeological topsoil relicts and the sampling strategy can be found in Lauer *et al.* [8].

Because of the complexity of the investigated proxies, this study focused on ten selected slot pits (n = 14 soil samples), six selected pits of pit alignments (n = 6 soil samples) as well as one Early Weichselian humic zone (Table 1). The Early Weichselian humic zone (i.e. “Humuszone”: dark humic-rich paleosol, which is considered to be formed in an interstadial period during the Early Weichselian; [55]), was located in Düren Arnoldsweiler (Lower Rhine Basin, Western Germany). Thus, this paleosol is assumed to represent the soil status before the onset of farming activity. It may therefore be assumed that the respective sample received if at all, only sparse amounts of wild-life faeces but no significant amounts of animal manure, like manured fields do. Thus, this paleosol served as a reference for the comparison of arable vs. natural soil and is designated as “reference soil” in the following. A comparable humic zone in Garzweiler/Elsbachtal also located in the Lower Rhine Basin (ca. 30 km distance apart) was dated to 87,100±8,300 BP [56]. Hence, the investigated humic zone of Düren Arnoldsweiler is considered to be of the same age.

The subsoils (n = 20 soil samples) were sampled in the same depth, but at short lateral distances from the pit fillings (<0.5 m). Moreover, we sampled recent topsoils (n = 7 soil samples). These samples served as control against post-depositional translocation processes. It should be noted, though, that along small distances we cannot fully discount the possibility that there was a lateral transport of any of the substances under study from the pit filling into the adjacent subsoil as well as vice versa (e.g. via water or soil dwelling animals). For the same reasons, also a vertical transport into the subsoil from upper soil layers by the same mechanisms cannot be ruled out. Hence, we did not expect to find zero biomarker and element contents for all parameters in the subsoils. The pit fillings and adjacent subsoils, however, were deep buried features (on average with a depth of 1.60 m). Additionally, we assumed that vertical leaching with convective-dispersive flow from the recent topsoil is unlikely for the majority of the studied, more or less immobile soil constituents (P, some micronutrients, peptide-bound amino acids, steroids, black carbon; see also Lauer *et al.* [8]).

### 2 Basic soil properties

Prior to laboratory analyses, all samples were dried and sieved to <2 mm. Sub-samples were ball-milled. For all samples the total carbon, nitrogen, and carbonate contents, as well as pH-values (CaCl_2_) were determined according to [57]–[59]. The grain size distribution was determined in selected samples (n = 24) on the basis of the sedimentation and pipette method by Köhn [60].

### 3 Micronutrient analysis

The micronutrient contents were determined after *aqua regia* digestion [61] of dried and sieved soil samples with subsequent quantification via inductively coupled plasma optical emission spectrometry (ULTIMA 2 ICP-OES spectrometer, HORIBA, Japan). Besides the total P content, the present analyses concentrated on the following elements: iodine (I), copper (Cu), manganese (Mn), molybdenum (Mo), selenium (Se) and zinc (Zn), in addition we assessed the contents of Ca, Mg, S, Fe, as well as heavy metals like Co, Ni, Ti, Sr, Zr (see Table S1). The selected micronutrients also are typical essential trace elements for human nutrition (I), crops (Mo) or both (Cu, Mn, Se, Zn) [62], [63].

### 4 Stable N isotope analysis

Stable isotope analysis of milled bulk soil samples was carried out by dry combustion in an elemental analyzer (Flash EA, 1112 Series, Thermo Fisher Scientific GmbH, Bremen, Germany) coupled with a Delta V Advantage isotope ratio mass spectrometer (Thermo Fisher Scientific GmbH, Bremen, Germany). Nitrogen isotopic values are expressed in δ^15^N relative to the isotopic composition of air (reference standard): 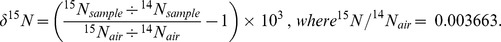



Calibration was performed using acetanilide (δ^15^N: 1.15‰) and ammonium sulfate (δ^15^N: 20.3‰) as certified standards (purchased from the International Atomic Energy Agency, Vienna, Austria). Precision of analyses was ±0.15‰ for duplicate measurements.

### 5 Amino acid analysis

Amino acid enantiomers were determined in two replicates by the method of Amelung and Zhang [64]. As microorganisms are known to synthesize a variety of D-amino acids in free and water-soluble forms [65], free amino acids were removed with 1 M HCl (12 h, 25°C; [66]). The remaining soil was hydrolyzed with 6 M HCl (12 h, 105°C), the solution was filtered, purified via cation exchange resins using 0.1 M oxalic acid for metal complexing, eluted with 2.5 M NH_4_OH, and dried on a rotary evaporator. Amino acid enantiomers were converted into N-pentafluoropropionyl-amino acid isopropyl esters as described by Frank *et al.* [67]. Gas chromatographic separation of the amino acid derivatives was carried out on an Agilent 6890 gas chromatograph with mass-spectrometer, using a chiral column (Chirasil-L-Val, 25 m×0.25 mm; Agilent Technologies GmbH, Böblingen, Germany) and electron impact ionization. For recovery assessment L-norvaline (internal standard 1) was added after hydrolysis and calculated relative to D-methionine (internal standard 2), which was added before derivatization. Recovery of L-norvaline averaged 83±18% (mean ± standard deviation). Arginine, cysteine, histidine, and tryptophan cannot be measured with this method. Hydrolysis transforms glutamine and asparagine, if present, into their carboxylic acids; so we report concentrations of glutamic acid and aspartic acid. The total amino acid concentration was calculated as the sum of the D- and L-enantiomers.

### 6 Steroid analysis

The steroid contents were determined according to the method of Birk *et al.* [49] with slight modifications (Fig. S1; see following section for method details). Due to the complex method only selected samples (n = 13 soil samples) of two excavation sites in Western Germany (i.e. Merzenich and Düren Arnoldsweiler) were analyzed - in order to compare samples from one archive with the same history of pedogenesis.

#### 6.1 Steroid extraction, separation and derivatization

Steroid analyses followed the protocol of Birk *et al.* [49] (see also Fig. S1) with modifications concerning sample extraction and quantification. In brief, for each sample 10 g of milled soil were spiked with 5β-pregnan-3α-ol-20-one, 5β-pregnan-3α-ol, and isodeoxycholic acid as recovery standards and extracted subsequently with dichloromethane/methanol (2∶1, v/v) and dichloromethane/methanol (1∶3, v/v), using accelerated solvent extraction (ASE Dionex 350; at 100°C). The dried total lipid extracts (TLE) were saponified with 5% KOH in methanol (10–14 h). Afterwards, the extracts were separated into a neutral fraction (including the sterols, stanols and stanones) and an acidic fraction (including the bile acids) by a repeated liquid-liquid extraction with chloroform (3×15 mL), an acidification with 1 M HCl (pH≤2), and a further liquid-liquid extraction with chloroform (3×15 mL).

After evaporation and drying of the extracts, the neutral fraction was separated by solid phase extraction (SPE) using 5% deactivated silica gel and (i) 5 mL hexane (for preconditioning), (ii) 5 mL hexane, (iii) 3 mL dichloromethane and (iv) 2 mL dichloromethane/acetone (2∶1, v/v). The second fraction eluted with hexane was discarded; the third and fourth fraction were combined and dried. The acidic fraction was methylated (addition of 1 mL dry 1.25 M HCl in methanol and heating at 80°C for 2 h; then addition of 1 mL Millipore water with following repeated liquid-liquid-extraction with 3×1 mL hexane) and separated in a fatty acid and a bile acid fraction by SPE. Therefore, activated silica gel was used with (i) 5 mL hexane/dichloromethane (2∶1, v/v) (for preconditioning), (ii) 4 mL dichloromethane/hexane (2∶1, v/v) and (iii) 5 mL dichloromethane/methanol (2∶1, v/v). The second eluted fraction was discarded, the third fraction, containing the bile acid methyl esters, was dried.

The sterol, stanole, and stanone extracts were silylated adding Sylon as derivatization reagents and heating at 70°C for 1 h, the bile acid methyl ester extracts by adding 50 µl toluene and *N,O*-bis(trimethylsilyl)trifluoroacetamide (BSTFA) containing N-trimethylsilylimidazole (TSIM) (98∶2, v/v) as derivatization reagents and heating at 80°C for 1 h.

#### 6.2 Steroid measurements and quantification

As a second internal standard α-cholestane (in toluene) was added to both fractions (the bile acid methyl ester as well as the sterol, stanol, and stanone fraction) and finally the samples were analyzed using gas chromatography-mass spectrometry (GC/MS) with an Agilent 5973 quadrupole mass spectrometer coupled to an Agilent 6890 gas chromatograph. Gas chromatographic separation of the steroids was carried out with an Optima-5 MS column, including a 10 m pre-column (40 m×0.25 mm×0.25 µm; Macherey-Nagel, Düren, Germany) and electron ionization. The injection port was set to 250°C and samples were injected in splitless mode. For the sterol, stanol and stanone separation the column temperature program was: 80°C (1.5 min) to 265°C at 12°C min^−1^, to 288°C at 0.75°C min^−1^, to 300°C at 10°C min^−1^ (held 12 min), and to 340°C at 25°C min^−1^ (held 5 min). For the bile acid separation the column temperature program was: 80°C (1.5 min) to 265°C at 12°C min^−1^, to 288°C at 0.75°C min^−1^, to 300°C at 10°C min^−1^ (held 12 min), and to 340°C at 25°C min^−1^ (held 5 min). Scan mode and the comparison with external standards were used to verify peak identity; measurements in selected ion monitoring mode (SIM) were carried out for quantification. Table S2 shows the steroid structures, the retention times, and the selected characteristic ion fragments.

The quantification of steroids was done using an external standard series (with the relevant, commercially available steroids in five concentrations) with sample matrix for each sample. For sterols, stanols, and stanones the recovery of the first internal standard pregnanolone (5β-pregnan-3α-ol-20-one) averaged 83±21% (mean ± standard deviation), and for bile acids the recovery of isodeoxycholic acid averaged 56±20% (mean ± standard deviation). The limit of quantification was 5 µg kg^−1^ soil for coprostanol, epi-coprostanol, and deoxycholic acid and 10 µg kg^−1^ soil for all other steroids.

### 7 Black carbon analysis

Black carbon analyses were conducted using the benzene polycarboxylic acid (BPCA) method as described by Glaser *et al.* [68] with revisions from Brodowski *et al.* [69]. The samples (two replicates) were first treated with 4 M trifluoroacetic acid (TFA) to remove polyvalent cations and then digested with HNO_3_ at 170°C for 8 h to yield benzene acids with different degree of carboxylation (for the structure of black carbon and benzene polycarboxylic acid degradation products see scheme in Kögel-Knaber and Amelung [70]). After cleanup via cation exchange resin (Dowex 50 W×8, 200–400 mesh, Fluka, Steinheim, Germany), the samples were silylated and BPCAs were measured using gas chromatography with flame ionization detection (GC-FID; Agilent 6890 gas chromatograph; Optima-5 column; 30 m×0.25 mm; Supelco, Steinheim, Germany). For recovery assessment citric acid (internal standard 1) was added prior to the cleanup step and calculated to biphenyl dicarboxylic acid (internal standard 2), which was added to the samples prior to derivatization. Carefully monitoring the pH avoided decomposition of citric acid during sample processing (as criticized by Schneider *et al.* [44]); the recovery of the first internal standard averaged 67±1% (mean ± standard deviation; optimal recovery is >70%). Results are given in the following as the carbon content of BC (BC-C) corrected for loss during sample preparation via internal standard 1 and a conversion factor of 2.27 and normalized to SOC (g BC-C per kg SOC).

### 8 Color measurements

Dried and homogenized fine-earth samples (<2 mm) were measured in triplicates using a spectrophotometer (CM-5; Konica Minolta, Japan). Reflected light was detected under standardized observation conditions (2° Standard Observer, Illuminant C), and color spectra were obtained in the 360 to 740 nm range, in 10 nm increments. The spectral information was converted into the CIELAB Color Space (L*a*b*; CIE 1976) using the Software SpectraMagic NX (Konica Minolta, Japan). The L* values indicate lightness as the extinction of light, or luminance, on a scale from L* 0 (absolute black) to L* 100 (absolute white).

### 9 Statistical evaluation

Differences in geochemical parameters between pit filling, adjacent subsoil and recent topsoil were evaluated using STATISTICA (8.0 for Windows; Statsoft Europe GmbH, Hamburg, Germany). For the comparison of normally distributed data, we applied t-tests for paired samples, since the properties of the pit filling were directly related to those of the adjacent subsoil and recent topsoil. The Wilcoxon-Rank-test was used for data that did not pass the test of normal distribution (Shapiro-Wilk-Test). The differences between the soil regions were evaluated by a Student's t-test. The correlation between the amino acid contents and the SOC and total N as well as the correlation between the pH-values and D-lysine contents were determined using the Spearman rank correlation. Also the correlations between the SOC contents, the black carbon contents and the L* values were determined using the Spearman rank correlation. Significance was set at *p*<0.05 unless otherwise stated.

## Results and Discussion

### 1 Nutrient status of archaeological topsoil relicts

In this study, we analyzed key elements in arable soil relicts outside ancient settlements in order to gain more information on nutrient contents as they potentially affect plant growth (e.g. P, micronutrients) and human nutrition (e.g. I and Se). We presumed that if a nutrient was deficient, it should be depleted in the prehistoric topsoil relative to its adjacent subsoil, because of significant, continued root uptake of nutrients from topsoils and less intensively from subsoils. The results the *aqua regia* extractable contents of total P and of the micronutrients I, Cu, Mn, Se, and Zn of the pit fillings in the Luvisol and Phaeozem regions could not show a depletion compared with the respective adjacent subsoils, with one exception for the P content in one site in the Chernozem region (i.e. Jüdendorf; Table 2). The pit fillings of this site were also not enriched in extractable P when compared with the adjacent subsoils [8].

**Table 2:** Contents of selected *aqua regia* extractable elements (in mg kg^−1^ soil) in the pit fillings, adjacent subsoils and recent topsoils of the Chernozem and Phaeozem region.

Region/ excavation site	Sample type	n^a^	pH^b^		P		I		Cu		Mn		Mo		Se		Zn	
					[mg kg^−1^ soil]													
**Central Germany**																		
**Chernozem region**																		
Kleingräfendorf	pit filling	2	8.1		388	(348–428)	76	(0–151)	9	(5–13)	399	(388–410)	1	(0–2)	4	(0–8)	31	(25–37)
	subsoil	2	8.2		377	(337–416)	75	(0–150)	6	(3–9)	282	(246–318)	1	(0–2)	4	(0–7)	20	(19–21)
	topsoil	1	7.6		683		216		16		490		1		9		44	
Oechlitz	pit filling	1	8.1		873		667		12		421		2		8		37	
	subsoil	1	8.2		843		398		9		316		2		8		27	
	topsoil	1	7.7		1339		645		15		476		3		10		42	
Jüdendorf	pit filling	3	7.8		354	(37)	96	(30)	15	(6)	453	(42)	1	(0.6)	3	(3)	39	(5)
	subsoil	3	7.8		386	(9)	65	(9)	11	(4)	397	(48)	0	(0.1)	0		38	(5)
	topsoil	3	7.3		423	(21)	44	(21)	17	(5)	512	(41)	0	(0.2)	0		57	(5)
*Mean*	*pit filling*	*6*	*7.9*	*ab*	*452*	*(87) a*	*185*	*(99) a*	*13*	*(3) a*	*430*	*(22) a*	*1*	*(0.5) a*	*4*	*(2) a*	*36*	*(3) a*
	*subsoil*	*6*	*8.0*	*ab*	*459*	*(78) a*	*124*	*(58) b*	*9*	*(2) b*	*345*	*(33) a*	*1*	*(0.4) a*	*2*	*(2) a*	*31*	*(4) a*
	*topsoil*	*5*	*7.5*	*b*	*641*	*(178) a*	*211*	*(117) ab*	*16*	*(3) ab*	*505*	*(24) b*	*1*	*(0.5) a*	*4*	*(2) a*	*50*	*(4) b*
**Phaeozem region**																		
Prießnitz	pit filling	2	6.5	a	593	(513–674) a	229	(138–320) a	13	(13–13) a	859	(511–1207) a	1	(0–1) a	10	(10–11) a	58	(54–62) a
	subsoil	2	7.5	a	375	(322–427) a	133	(76–190) a	14	(13–16) a	1220	(468–1972) a	2	(1–2) a	10	(9–11) a	41	(35–47) a
	topsoil	1	5.3	b	587	a	270	a	13	a	652	a	1	a	10	a	43	a

Means with standard errors in parentheses and for n = 2 each value is listed in parentheses. Within one region, different letters designate significant (*p*<0.05) differences between sample types.

It is noteworthy that the contents of some micronutrients in the pit fillings of all studied soil regions were similar to those in recent fertilized topsoils (Table 2 and Table 3). Similarly, also the contents of other nutrients like Ca, Mg, S, and Co did not point to significantly lowered nutrient stocks in the prehistoric topsoils when compared with recent topsoils (Table S1). Generally, the plant availability of many micronutrients depends on the soil's pH. The pH values of prehistoric topsoils were probably higher than today, because decalcification with subsequent chemical weathering on the primary calcareous loess (Upper Weichselian) did not start before the Late Glacial (see also Schlummer *et al*., [71]). The micronutrients Cu, Mn, and Zn are immobilized in calcareous soils [72], [73], therefore, their available portion in soil might have been small in prehistory despite of their elevated total contents. These micronutrients possess a low mobility under these conditions of relatively high pH values, because they form carbonate complexes or insoluble oxides. Thus, these elements may have been stored for long periods in a soil depth, where acid root exudates may not have found their way to mobilize them [74]–[76]. On the contrary, the availability of I, Mo, and Se usually increases with increasing soil pH, suggesting that these elements were likely even more available to plants in prehistory than they are nowadays.

**Table 3:** Contents of selected *aqua regia* extractable elements (in mg kg^−1^ soil) in the pit fillings, adjacent subsoils, recent topsoils and Early Weichselian humic zone of the Luvisol region.

Region/excavation site	Sample type	n^a^	pH^b^		P		I		Cu		Mn		Mo		Se		Zn	
					[mg kg^−1^ soil]													
**Western Germany**																		
**Luvisol region**																		
Merzenich	pit filling	9	7.0		831	(38)	345	(47)	17	(1)	780	(55)	2	(0.2)	13	(1.1)	66	(7)
	subsoil	11	7.3		669	(69)	51	(28)	17	(1)	862	(125)	3	(0.2)	9	(0.8)	46	(3)
	topsoil	1	6.8		1225		12		15		652		3		7		66	
Pulheim	pit filling	2	6.4		836	(817–856)	118	(0–236)	20	(14–27)	505	(427–583)	1	(0–3)	5	(0–11)	52	(43–61)
	subsoil	2	6.4		733	(725–740)	97	(0–194)	18	(12–24)	516	(508–524)	1	(0–3)	5	(0–11)	60	(40–80)
	topsoil	1	5.3		800		207		11		627		2		7		52	
*Mean*	*pit filling*	*11*	*6.9*	*a*	*832*	*(36) a*	*310*	*(52) a*	*18*	*(1) a*	*737*	*(52) a*	*2*	*(0.3) a*	*12*	*(1) a*	*64*	*(6) ab*
	*subsoil*	*13*	*7.2*	*b*	*680*	*(63) b*	*59*	*(28) b*	*17*	*(1) ab*	*799*	*(120) a*	*3*	*(0.3) ab*	*8*	*(1) b*	*49*	*(4) ab*
	*topsoil*	*2*	*6.0*	*c*	*1013*	*(213) c*	*110*	*(98) b*	*13*	*(2) b*	*639*	*(12) a*	*3*	*(0.3) b*	*7*	*(0.1) c*	*59*	*(7) b*
Düren Arnoldsweiler	humic zone	1	6.6		257		109		13		767		1		10		39	

Means with standard errors in parentheses and for n = 2 each value is listed in parentheses. Within one region, different letters designate significant (*p*<0.05) differences between sample types.

a number of samples,

b in 0.01 CaCl_2._

In summary, the analyses do not support the hypothesis that there might have been ancient P or micronutrient deficiencies in the studied Neolithic and Bronze Age arable soils due to agricultural land use. Yet, it remains unclear whether there have been nutrient replacements, e.g. in form of N fixation or organic fertilization.

### 2 Proxies for prehistoric nitrogen input

#### Stable N isotope signature

**Figure 1:**
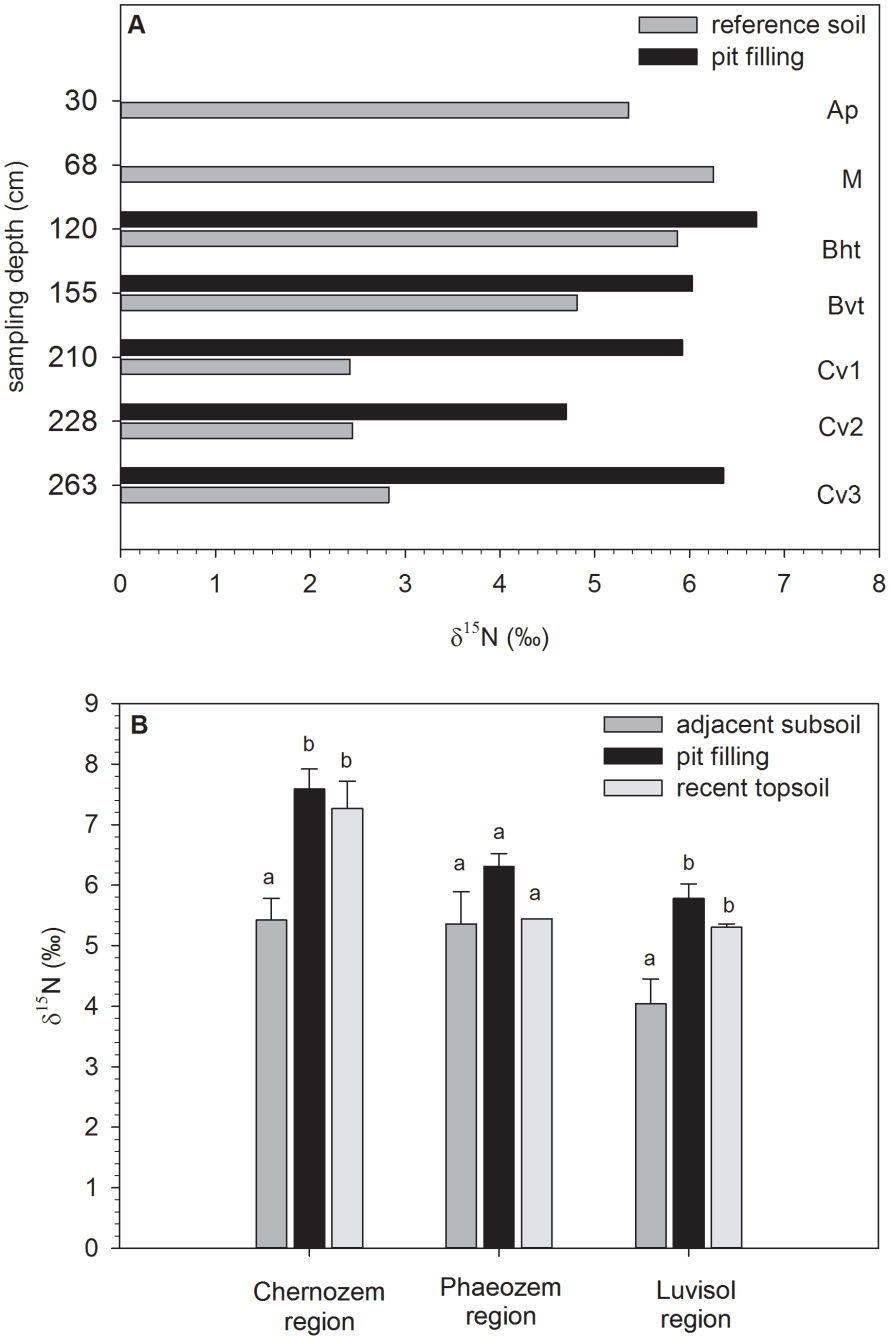
Depth distribution of δ^15^N (in ‰) at the excavation site Merzenich in the Luvisol region in the reference soil outside the pit, and in the pit filling (A). Soil horizon designation of reference soil follows the German soil classification [132]. Ap = ploughed humic horizon, M = colluvial deposit, Bht = humic, argic horizon, Bvt cambic, argic horizon, Cv = parent loess, slightly weathered. And δ^15^N values (in ‰) in the adjacent subsoil, the pit filling representing the prehistoric topsoil, and recent topsoil in the Chernozem, Phaeozem, and Luvisol region (B). The error bars represent the standard error. Within one region, different letters designate significant (*p*<0.05) differences between sample sets.

Neolithic agriculture included cropping of legumes as shown by archaeobotanical analyses [77]. Nitrogen fixing plants, like legumes, adapt to the isotopic signature of N_2_ in the air, and their δ^15^N value approaches zero [20]–[22]. However, the increased abundance of legumes usually showed little if any bulk soil δ^15^N discrimination during N_2_ fixation [46], [47], [78]. Here, the recent topsoil of one excavation site (Merzenich; Luvisol region in Western Germany) showed δ^15^N values of 5.9‰ (Fig. 1A). The slot pit was covered by a colluvial soil horizon (“M”; 38 cm thick), which exhibited larger δ^15^N values (6.7‰; Fig. 1A). The δ^15^N values of the slot pits ranged from 4.7‰ to 6.7‰. These values are far off typical values that were found in fields that were cropped with N fixing plants – hence, if there has been a legume cropping, it did not occur long enough to significantly influence the soil δ^15^N values.

Recent native grassland whose vegetation frequently contains N fixing clover, usually also shows low soil δ^15^N values close to zero, as well (in the range of −1‰ to +2‰, but not larger than 5‰) [47], [79]–[81]. According to these δ^15^N values, the prominence of a native grassland vegetation in the studied regions and time periods can be excluded. Also recent forests usually exhibit δ^15^N values between −2.8 and +2‰ under comparable climatic conditions [82], [83]. Hence, the large δ^15^N values found in our study give strong support to the assumption that the pit filling material was neither grassland nor forest soil but, in consequence, arable land. However, also the Early Weichselian humic zone located in boreal vegetation [84] before farming activity exhibited δ^15^N of 5.3‰. But as it is reported that also the soil age exerts an influence on ecosystem δ^15^N values over longer timescales with increasing δ^15^N values in older soils [82], [85]. Thus, the humic zone as the oldest investigated soil horizon (i.e. Early Weichselian) might have been altered during the time of deposition. This aging effect might have also been the cause for a slight increase of the δ^15^N values of the pit fillings.

The subsoil outside the pit, exhibited significantly smaller δ^15^N values (2.4–5.9‰) than the pit filling material (4.7–6.7‰), showing even lower values in the deeper loess dominated horizons (i.e. C-horizons) than the overlying reference soil horizons (Fig. 1a). This is remarkable, since δ^15^N values of soils usually increase with soil depth or with decreasing organic N contents because the processes of soil organic nitrogen formation and loss result in an enrichment of the heavy isotope and hence, in larger δ^15^N values [47], [78]. Hence, we have to assume that the overlying material, particularly the recent Ap horizon as well as the prehistoric A horizon-material within the pits, was influenced by external N input. Typical inputs showing elevated δ^15^N values are organic manures. During storage of organic manures usually large losses of the light ^14^N occur by ammonia volatilization, resulting in δ^15^N values of 5‰ or even larger [26], [47], [81], [86]. The elevated ^15^N ratios in the pit fillings could thus be seen as a first indicator of external nitrogen input caused by prehistoric manuring [47], [86]–[88]. However, isotope ratios are usually not used as direct biomarkers for manure, hence, we added analyses of steroids, for a more specific indication (see below).

When comparing the three soil regions (i.e. Chernozems, Phaeozems and Luvisols) no change in δ^15^N pattern can be observed: all pit fillings exhibited large δ^15^N values (5.8–7.6‰) with no significant differences to the values of the respective recent topsoils (5.3–7.3‰). The material of the pit fillings was thus also enriched in ^15^N compared with that of the adjacent subsoils (Fig. 1B). Hence, the δ^15^N values reported for all archaeological soils of this study appear to be typical for larger soil areas.

Nevertheless, the level of the δ^15^N values differed: Chernozem pit fillings in Central Germany exhibited on average significantly larger δ^15^N values (7.6±0.3‰ standard error) than the Luvisol pit fillings in Western Germany (5.6±0.2‰) (Fig. 1B). The reasons for these divergences can be manifold: either there were different N-inputs [81], [89], varying attributes of the soil (e.g. microbial population, mineralogical composition), or different pedogenetic processes. All of them probably contribute to a different degree of δ^15^N fractionation [78], [90]–[92].

#### Amino acid signature

The amino acid composition may be specific for distinct crops [23]–[25], hence, we analyzed their composition in archaeological soil samples to elucidate the sources of organic N inputs in prehistory. The total contents of amino acids (i.e. the sum of D- and L-enantiomers of the respective amino acids under study) correlated closely with SOC (r = 0.72) and total N contents (r = 0.87), which is in accordance with the results of other studies [93]–[95]. For a better understanding of the role of amino acids in SOM dynamics, the amino acids were expressed in grams per kilogram total N. The element-normalized amino acid contents then amounted to 334–1734 g kg^−1^ N (Table 4). Generally, the soil amino acids contain approximately 7–21% N. In the recent topsoils of our study, the amino acids explained 20–22% of total soil N irrespective of the study region (Table 4). Other studies reported similar proportions (i.e. 20–40% of total N; [96], [97]. Contrastingly, the content of the amino acid-N of the pit fillings and the adjacent subsoils corresponded only to 5–16% of the contents of soil N (Table 4), which is significantly lower than the amino acid-N to soil N contribution in the recent topsoils. With increasing soil depth and increasing soil age the proportion of non-hydrolysable N and undefined N increased. This indicates either that older SOM in the pit fillings and in the subsoils comprised a larger fraction of chemically stable peptide N or a selective decomposition of hydrolysable amino acids in the pit fillings and subsoils. Similarly, Mikutta *et al.* [98] found a larger proportion of non-hydrolysable amino acids across a long-term chronosequence in Hawaii, indicating a preferential accumulation of mineral-associated organic N with time (up to 4,100 years) and with soil depth. Equally, Glaser [99] revealed that the amino acid-N pool of Amazonian dark earths only contributed to 18–25% of total N, and assumed that a majority of these unknown N-pools were present in heterocyclic compounds. In any case, due to the low amount of amino acid-N recovered in the ancient pit fillings, the usefulness of the amino acids in identifying different soil organic nitrogen (SON) sources from prehistoric crops was limited, irrespective of possible transformations of the amino acid pool during SON genesis. Nitrogen that is introduced into the soil by legume cultivation can be detected due to the involved increase of hydrolysable amino acid contents [96], [100]. The amino acid contents of the pit fillings were small compared to the recent topsoils, therefore, the amino acid analyses could not reveal an evidence for legume cultivation in the prehistoric arable topsoils.

**Table 4:** Contents of total N and amino acid as well as respective contributions and selected amino acid ratios in the pit fillings, adjacent subsoils, and recent topsoils.

Region/excavation site	Sample type	n^a^	total N [g kg^−1^]		amino acids [g kg^−1^ N]		AA-N/N [%]		D/L alanine		D/L lysine		L-threonine/L-lysine	
**Central Germany**														
***Chernozem region***	pit filling	*6*	0.5	(0.0) a	1121	(64) a	14.1	(0.8) a	0.18	(0.01) a	0.04	(0.003) a	1.0	(0.10) a
	subsoil	*6*	0.2	(0.1) a	1238	(107) a	15.5	(1.4) a	0.15	(0.01) ab	0.04	(0.004) a	0.9	(0.12) a
	topsoil	*5*	1.1	(0.2) b	1616	(67) b	20.2	(0.8) b	0.14	(0.01) b	0.03	(0.004) b	1.1	(0.13) b
														
***Phaeozem region***	pit filling	2	0.5	(0.1) ab	635	(11) a	8.1	(0.1) a	0.19	(0.01) a	0.06	(0.01) ab	0.7	(0.12) a
	subsoil	2	0.2	(0.1) a	635	(24) a	8.1	(0.4) a	0.18	(0.02) a	0.06	(0.002) a	0.6	(0.23) a
	topsoil	1	1.2	b	1694	b	21.1	b	0.12	a	0.03	b	1.4	a
**Western Germany**														
***Luvisol region***	pit filling	11	0.4	(0.0) a	334	(29) a	4.5	(0.4) a	0.20	(0.01) a	0.05	(0.002) a	0.5	(0.05) a
	subsoil	*13*	0.2	(0.0) b	345	(48) a	4.6	(0.6) a	0.17	(0.01) b	0.05	(0.002) a	0.6	(0.06) b
	topsoil	*2*	1.4	(0.6) c	1734	(183) b	22.0	(2.1) b	0.11	(0.02) c	0.02	(0.003) b	1.5	(0.05) c
Düren Arnoldsweiler	humic zone	1	0.4		362		4.9		0.21		0.06		0.3	

Means with the standard errors in parentheses. Within one region, different letters designate significant (*p*<0.05) differences between sample types.

a number of samples;

b contribution of amino acid-N to total N.

Yet, the amino acid contents differed between the sites. The Neolithic pit fillings of the Luvisol and Phaeozem region showed significantly smaller contents of amino acid-N compared to the Bronze Age pit fillings of the Chernozem region (Table 4). On the one hand this finding might reflect the higher age of SON that – similarly to the subsoils – resulted in smaller amino acid-N proportions. On the other hand this finding may be a result of significant larger clay contents in the Neolithic pit fillings of the Phaeozem region (averagely 31% clay in the pit fillings) and Luvisol region (averagely 24% clay), compared with the Bronze Age pit fillings (averagely 6% clay) located in the Chernozem region as higher clay contents can have negative impact on the extractability of amino acids [93], [101]. In both cases, the properties of the SON pool were apparently influenced by pedogenesis, suggesting that the amino acid method was not suitable biomarker for the origin of amino acids in our arable sites, as, e.g. previously found for compound-specific ^15^N amino acid signals in Bronze Age grassland sites [26].

Lacking clear evidence of legume traces does not mean that legumes were not cropped at all; such a conclusion would also have contradicted archaeobotanical evidence [77]. Looking into the amino acid composition of different crops, for instance, showed that the threonine to lysine ratio is typically around 0.6 for legumes but 1.0 for cereals [23]–[25]. Hence, smaller threonine-to-lysine ratios in soils might reflect an increasing cropping of legumes. We tested this by analyzing an arable soil that had been recently cropped for three years with clover (*Trifolium pratense*). Indeed this soil exhibited an enrichment of lysine relative to threonine with a L-threonine to L-lysine ratio of 0.6 (data not shown). Since D-threonine is also produced by microbes [102], the amino acid ratios were based on the respective L-types of these two amino acids. The recent topsoils of the study regions showed L-threonine to L-lysine ratios of 1.1 to 1.5 (Table 4), thus reflecting the dominance of cereal production in modern crop rotations. In contrast, the prehistoric topsoils (i.e. pit fillings) revealed L-threonine to L-lysine ratios of 0.5 in the Luvisol region, 0.6 in the Phaeozem region, and 1.0 in the Chernozem region (Table 4). These results would have been in line with observations of archaeologists that there was legume cultivation in prehistoric agriculture, even if it was not dominant [77], [103]. However, these data are not supported by low δ^15^N ratios and elevated amino acid N contents (see above). Moreover, also the adjacent subsoils showed L-threonine to L-lysine ratios of 0.6 to 0.9, indicating that this ratio might as well have been influenced by SOM genesis in the soil profiles besides legume-N input.

Amino acid enantiomers have been proposed as bacterial and age markers [94]. D-glutamic acid and D-alanine, being part of the bacterial cell wall, are enriched during SOM formation and transformation [94], [95], [98]. And indeed, we found more bacterial derived D-amino acids in the pit fillings and in adjacent subsoils than in the recent topsoils (Table 4; data for alanine). This finding suggests that larger proportions of the total SON pool had been cycled by bacteria in the pit fillings and subsoils than in the recent surface soils, i.e. the SOM of the prehistoric topsoils had been altered relative to the surface soils. Such processes may have contributed to changes in the amino acid composition as mentioned above, and they may in part explain the elevated δ^15^N values in the pit fillings above those in the recent surface soils. These findings do explain the general large soil δ^15^N values in comparison to the deeper subsoils.

Apart from using the contents of the D-enantiomers of glutamic acid and alanine as markers for the contribution of bacterial cell walls to SON, the D/L ratio of lysine has been discussed as a marker for an age assessment of the respective protein pool and of total soil organic matter because D-lysine seems to originate solely from abiotic cell aging [94]. Increasing D/L ratios of lysine therewith correlated with SOM age in other soils [94]. This marker confirmed our assumption that the SOM age of the pit fillings and the adjacent subsoils was significantly older than that of the respective recent topsoils (Table 4). This finding was valid for all studied soil regions. The Early Weichselian humic zone exhibited the highest D/L ratios indicating the oldest investigated soil sample. Comparing the studied geoarchives, the Bronze Age pits in the Chernozem region revealed significantly smaller D/L ratios of lysine, and therefore a younger age than the Neolithic pit fillings in the Phaeozem and Luvisol region (Table 4). However, a negative correlation of the D-lysine content and the pH value of the investigated soils (r = −0.76) could be determined. That is why the production of D-lysine might be hindered in the more alkaline soils in Central Germany in comparison to the more neutral soils in Western Germany. Thus, this finding is consistent with those of Amelung [94] and Amelung *et al.* [104], who reported that amino acid racemization only allows a relative SON dating for a given environment but no estimation of the absolute age of proteins.

In summary, there was a significant accumulation of total N in the Neolithic and Bronze Age pit fillings. This SON contained amino acids, i.e. peptide-like structures were preserved. The composition of amino acids was typical for legume cropping, however, also for advanced stages of microbial SON transformation and SOM genesis. Overall, a significant cropping of legumes in prehistoric agriculture could not be assured on the bases of geochemical proxies. Noteworthy, the prehistoric topsoils had significantly elevated δ^15^N values compared with the adjacent subsoils, and therefore pointed to the use of manure in prehistoric agriculture. As the soil δ^15^N values do not provide a direct proxy for the origin of manure, we analyzed steroids as specific markers for faecal residues.

### 3 Proxies for prehistoric faecal input

#### Sterols, stanols, and stanones

The use of manure can be elucidated by steroid biomarkers, including sterols, stanols, and stanones ([30], [49], [105] see also Table S2).

All selected Neolithic soil relicts of the Lower Rhine Basin and the respective adjacent subsoils exhibited only little if any quantifiable amounts of cholesterol (16–94 µg kg^−1^) and the plant-derived sterol β-sitosterol (11–40 µg kg^−1^; Table 5). Stigmasterol, also a plant-derived sterol, could not be quantified or even not be determined in all studied samples. 5β-stigmastanol could only be detected in small amounts in one pit filling (11 µg kg^−1^). Similarly, coprostanol was not found in any of the studied samples. All reference soils including the adjacent subsoils ( = control) and the Early Weichselian humic zone (representing the soil before any farming activity) did not exhibit these faecal markers (5β-stanols) (Table 5).

**Table 5:** Contents of sterols and stanols (in µg kg^−1^ soil) of selected pit fillings, the respective adjacent subsoils ( = control) and the Early Weichselian humic zone ( = reference).

			Sterols					β-Stanols		α-Stanols		Epi-5β-Stanols			Sum	
			[µg kg^−1^ soil]													
Region/excavation site	Sample type	Soil depth [cm]	Chole- sterol*		β-Sito- sterol		Stigma- sterol	Copro-stanol*	β-stigma-stanol		α- Cholestanol*	α-Stigma-stanol		Epi-coprostanol		Σ sterols and stanols
**Western Germany/ Luvisol region**																
***Merzenich***	pit filling 1	123	n.q.		15.8		n.q.	n.q.	n.q.		n.d.	71.3		n.d.		87.1
	adjacent subsoil 1	123	61.8	(19.8)	26.8	(8.9)	n.q.	n.q.	n.d.		n.d.	95.5	(15.4)	n.d.		184.1
	pit filling 2	125	41.1	(7.0)	10.5		n.q.	n.q.	n.q.		n.d.	72.2		n.d.		123.8
	adjacent subsoil 2	125	51.2		37.6		11.1	n.d.	n.d.		n.q.	14.1		n.d.		102.9
	pit filling 3	130	43.0		40.0		10.1	n.q.	n.q.		n.d.	64.1		n.d.		157.2
	adjacent subsoil 3	130	16.3	(2.3)	20.7	(5.9)	n.q.	n.d.	n.d.		n.d.	67.1	(17.3)	n.d.		104.0
	pit filling 4	175	93.5	(31.5)	16.2	(7.0)	n.q.	n.q.	10.9		n.d.	62.0	(5.0)	n.d.		182.6
	adjacent subsoil 4	175	10.7	(8.8)	n.q.	(1.2)	n.q.	n.d.	n.d.		n.d.	13.7	(3.4)	n.d.		24.4
	pit filling 5	180	n.q.	(6.5)	14.0	(0.2)	n.q.	n.d.	n.d.		n.q.	30.3	(6.6)	n.d.		44.3
	adjacent subsoil 5	180	n.q.		n.q.		n.q.	n.d.	n.q.		n.q.	30.8		n.d.		30.8
	pit filling 6	263	10.9	(8.3)	n.q.	(2.3)	n.d.	n.q.	n.d.		n.d.	15.3	(11.7)	n.d.		26.3
	adjacent subsoil 6	263	n.q.		n.q.		n.q.	n.d.			n.d.	16.9	(6.3)	n.d.		16.9
***Düren Arnoldsweiler***	humic zone	170	n.q.		n.q.		n.d.	n.d.			n.d.	n.q.		n.d.		0.0

Standard deviation of the laboratory replicates is given in parentheses.

n.q. detectable, but not quantifiable (under quantification limit; 5 µg kg^−1^ soil for coprostanol and epi-coprostanol, and 10 µg kg^−1^ soil for all other steroids); n.d. not detectable (under detection limit);

*mark mainly animal-derived steroids. The sources of the other unmarked steroids are plant litter, root exudates, and faeces (of herbivores and omnivores).

Without a positive proof of detectable amounts of 5β-stanols (coprostanol or β-stigmastanol) in the studied Neolithic arable soils, except for pit filling 4, stanol analyses did not provide evidence that faeces had been deposited on the prehistoric topsoils. This is in contrast to earlier studies, which found 5β-stanols in approximately 3,500 years old Minoan terraces [106] and even in 10,000 years old stratified shell middens [107]. In this study, relatively large contents of α-stigmastanol could be determined in the pit fillings as well as in the adjacent subsoils (Table 5). This finding points to the larger natural abundance of α-stanols in soils due to their production in course of the transformation of their precursors, like – in the case of α-stigmastanol – the plant sterols β-sitosterol and stigmasterol (see Fig. S2) [108], [109].

In the studied soil relicts and the reference soils, stanones (5α-cholestan-3-one and 5β-cholestan-3-one) could only be detected below the limit of quantification (i.e. 10 µg kg^−1^; data not shown). Hence, also this third steroid group did not help to unambiguously detect prehistoric faecal inputs into the soils.

#### Bile acids

Bile acids are additional markers for faecal matter from vertebrates as they are formed from cholesterol in their liver and excreted in small amounts [30]. Combining bile acid and β-stanol analyses may even help to distinguish between the source of faecal matter (human, ruminant, and porcine) [28], [30], [37]. Additionally, bile acids are presumed to be more stable against degradation in soils than stanols and stanones [29], [36], [110] and, thus, can be detected in soils thousands of years after the application of the faeces [110]–[112].

**Figure 2:**
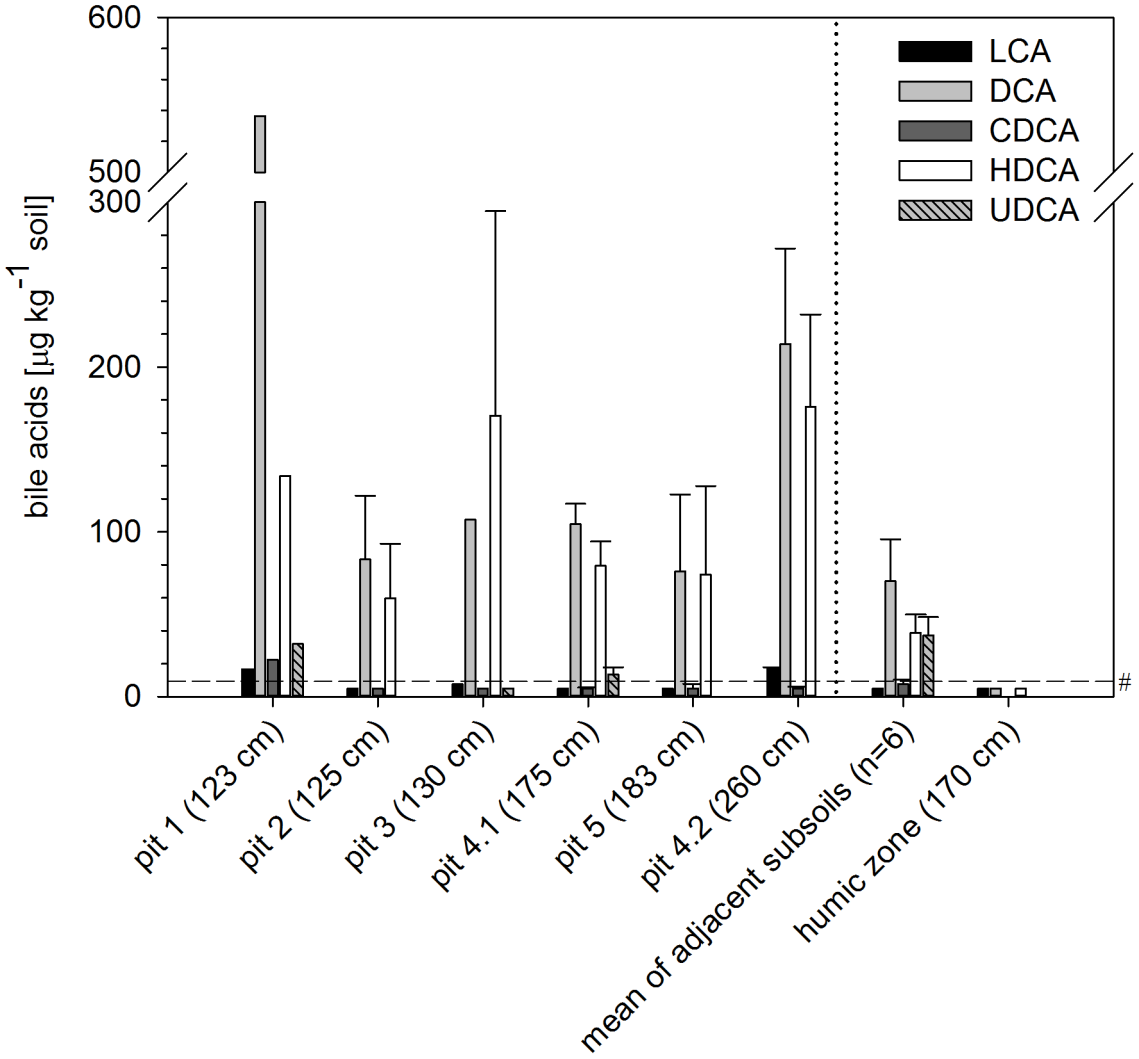
Bile acid contents of the selected pit fillings, the adjacent subsoils and the Early Weichselian humic zone at various soil depths as indicated in parentheses. The dashed line marks the limit of quantification. LCA = lithocholic acid, DCA = deoxycholic acid, CDCA = chenodeoxycholic acid, HDCA = hyodeoxycholic acid, UDCA = ursodeoxycholic acid. Error bars represent the standard deviation of laboratory replicates, whereas error bars of the mean of adjacent subsoils show the standard error of the six averaged adjacent subsoils.

All studied Neolithic pit fillings revealed larger contents of deoxycholic acid (DCA) and of hyodeoxycholic acid (HDCA) than the adjacent subsoils, in which only small contents of DCA and HDCA were detected (Fig. 2; see Table S3 for all data of the bile acids). There were little if any detectable amounts of other bile acids (i.e. lithocholic acid, chenodeoxycholic acid and ursodeoyxcholic acid) in the pit fillings and the adjacent subsoils. The Early Weichselian humic zone which represented the soil before any human farming and manuring activity, therefore, served as a reference sample without any anthropogenic inputs of bile acids and indeed showed no contents of bile acids above the quantification limit. These findings confirm the current assumption that beside faeces of most vertebrates there are no other significant sources of bile acids in the environment [40]. Therefore, the very small natural background occurrence of bile acids in the Early Weichselian humic zone (here below quantification limit) can only originate from the sparse input of faeces from wildlife animals, and the low contents support the absence of manuring. Yet, we cannot fully exclude a degradation or aging of the bile acids since their input into the humic zone in view of the long period of time (90.000 years), as there are no studies that have examined bile acids in soil samples of this high age.

Different contents of DCA and HDCA have been used to differentiate between faeces of ruminants and omnivores: HDCA is a biomarker of pig faeces, with DCA being absent [30], [36]. In contrast, human and cow faeces are dominated by DCA and lithocholic acid (LCA), but with cow faeces containing only small amounts of LCA [30], [36], [37]. Provided that all bile acids were decomposed equally quickly, the signal in the pits comprising DCA and HDCA indicated a mixture of pig, human and/or ruminant faeces. Pig faeces contain HDCA (and nearly no DCA), but human and ruminant faeces both contain DCA. The remarkably low contents of LCA in all pit fillings exclude an input of human faeces, because these faeces generally contain high amounts of LCA [30], [36]. In contrast, an input of ruminant faeces is more likely as these faeces generally contain low amounts of LCA [30], [36]. Hence, the enrichment of bile acids in the prehistoric arable topsoils - relative to the adjacent soil samples and the Early Weichselian humic zone - reflect that these soils had received faeces, likely produced by pigs and ruminant animals. It is very unlikely that these compounds were leached from the above-lying surface soils, because (i) there are indications that faecal residues are not prone to leaching [113], and (ii) because we could detect these compounds even in very deep pit fillings (>2.5 m), but hardly in the subsoils and not at all in quantifiable amounts in the Early Weichselian humic zone. For other rather immobile substances like phosphates, there was also no indication for a leaching into this depth [8]. We postulate, therefore, that the prehistoric surface soils had received a mixture of different manures.

Earlier archaeological studies that had used bile acids as tracers for faecal inputs into soils and sediments had mainly focused on Roman or younger soil relicts, such as drainage channels for latrines or sediment-filled sewers [38], [105], [110]. The detection of bile acids in the studied Neolithic soil relicts, being approx. 4,000 to 6,000 years old, confirms the long-lasting stability of these compounds as suggested by Bull *et al.* [110].

All in all, the studied sites in the Lower Rhine Basin contained indicators for a faecal input into the Neolithic arable soils, mainly because all pit fillings exhibited larger bile acid contents than the respective adjacent subsoils and the Early Weichselian humic zone (representing the soil before any farming activity). Here, the data suggests that Neolithic arable fields had been fertilized with faeces or livestock manure. Whether this has been done intentionally - by manuring or by hazard during grazing - remains unclear.

### 4 Proxies for prehistoric burning events

#### Soil color and black carbon

All pit fillings were characterized by a dark color, similar to the color of the recent topsoil but different from that of the adjacent subsoils [8]. This optical feature initially pointed to the origin of the pit fillings from ancient topsoil. Beyond the optical impression, color measurements allowed the quantitative comparison of the different sample types. The L* values, indicating the extinction of light on a scale from L* 0 (absolute black) to L* 100 (absolute white), were similar for the topsoils and pit fillings (Table 6). This is in line with the elevated contents of SOC in the pit filling material (Table 6). When relating color values (L* values) to black carbon (BC) and SOC contents (see Table S4 for single site data), there was a stronger correlation to BC (r = −0.76; *p*<0.01) than to SOC contents (r = −0.52; *p*<0.01), suggesting that the soil color was mainly related to burned, aromatic C compounds represented by the BC contents [43], [114].

**Table 6:** Contents of soil organic carbon and black carbon as well as respective contributions and soil color expressed as lightness (L*) in the pit fillings, adjacent subsoils, recent topsoils and Early Weichselian humic zone.

Region/excavation site	Sample type	n^a^	C_org_		BC		BC-C		Color		B3CA		B4CA		B5CA		B6CA	
			[g kg^−1^]		[g kg^−1^ soil]		[% of C_org_]		L*		[% of total BC]							
**Central Germany**																		
***Chernozem region***	pit filling	6	8.5	(0.5) a	2.2	(0.3) a	25.2	(2.1) a	52	(1.5) ab	3.5	(0.3) a	20.1	(0.6) a	36.5	(1.1) a	39.9	(0.9) a
	subsoil	6	5.3	(0.8) a	0.6	(0.3) a	10.0	(2.8) a	61	(2.5) a	20.2	(16.0) a	34.0	(7.9) a	21.8	(4.7) b	24.0	(5.5) b
	topsoil	5	13.0	(1.8) b	2.4	(0.4) a	19.8	(2.7) a	47	(1.5) b	3.8	(0.3) a	20.3	(2.0) a	35.0	(1.0) ab	40.9	(2.2) a
***Phaeozem region***	pit filling	2	5.7	(1.8) ab	1.1	(0.1) a	29.1	(2.3) a	53	(2.4) ab	4.6	(0.3) a	24.2	(2.2) a	35.7	(1.0) a	35.6	(3.5) a
	subsoil	2	3.5	(1.0) a	0.2	(0.1) a	7.5	(5.2) a	64	(0.3) a	5.3	(0.2) a	42.0	(6.8) a	29.6	(8.5) a	23.1	(1.5) a
	topsoil	1	11.9	b	0.3	a	7.7	a	58	b	3.4	a	24.9	a	32.9	a	38.8	a
**Western Germany**																		
***Luvisol region***	pit filling	11	4.1	(0.5) a	1.6	(0.2) a	36.6	(4.2) a	54	(0.8) a	4.5	(0.3) a	26.5	(1.7) a	35.2	(1.4) a	33.7	(1.0) a
	subsoil	13	3.5	(0.5) a	0.3	(0.1) b	8.7	(2.9) b	57	(0.5) b	2.7	(0.2) b	38.2	(2.9) a	29.8	(1.9) a	29.3	(1.0) b
	topsoil	2	14.2	(5.0) b	1.4	(0.3) a	10.4	(1.9) b	55	(1.1) a	3.5	(0.4) b	20.6	(1.0) b	32.1	(3.8) a	43.8	(2.4) c
Düren Arnoldsweiler	humic zone	1	2.4		0.5		22.7		62		3.7		37.0		28.9		30.4	

Means with the standard errors in parentheses. Within one region, different letters designate significant (*p*<0.05) differences between sample types.

a number of samples;

b in 0.01 M CaCl_2_.

In all samples the BC contents correlated significantly with the SOC contents (r = 0.71) with the contribution of BC to SOC being significantly larger in the pit fillings (24–38% of SOC was BC) than in the recent topsoils (8–25% of SOC was BC). Also in absolute amounts, the pit fillings revealed significantly larger BC contents (in g kg^−1^ fine earth) than the adjacent subsoils but similar BC contents compared with the respective recent topsoils (Table 6).

Among the individual soil regions, the Luvisol pit fillings revealed the largest contribution of BC to SOC (38%), whereas BC proportions in the Chernozem pit fillings were significantly smaller (25% BC of SOC; Table 6). Nevertheless, the recent topsoils of the Chernozem region revealed significantly larger BC contributions to SOC than the Luvisol topsoils, similar to those of other regions of the world [106]. Apparently, there was a preferred input of BC into the material of the pit fillings (the prehistoric topsoils) of the Luvisol region, which supports the idea of external BC inputs by slash- and burn managing practices [4], [115]. Higher clay contents in the Luvisol region might have also intensified stabilization processes - like BC interactions with mineral phases – leading to larger BC to SOC contributions of the pit filling material of the Luvisol region compared with that of the Chernozem region [116]–[118].

In the reference sample (i.e. the Early Weichselian humic zone), BC comprised 22% of the SOC, although the absolute BC contents were relatively small compared to those of the pit filling samples (Table 6). The BC stored in the Early Weichselian humic zone was likely produced by natural fires during this epoch [119], [120], which was expected because fire occurs as a frequent natural disturbance in boreal forests [121]. Also Early to Middle Weichselian paleosol relicts from adjacent sites revealed BC to SOC proportions between 15–35%, which could be related to natural fires during this epochs, as well [122]. Eckmeier *et al.* [7] who investigated Neolithic slot pits in the Lower Rhine Basin concluded that the large BC proportions of SOC (36% BC-C of SOC) could be a result of vegetation fires ignited by man, as a burning of prevalent temperate deciduous forests without human impact is shown to be very unlikely [123]–[125]. Similarly, Kleber *et al.* [126] supposed anthropogenic fire in prehistoric agriculture due to BC contents of up to 13% of SOC in Neolithic soil relicts in Central Germany. Hence, an attribution to agricultural fires cannot be achieved alone via BC quantification, but needs to be supported by environmental (e.g. archaeobotanical) and chronological data.

Furthermore, the quality of BC can be used to gain information about the type of fire. Generally, BC from high temperature fire is characterized by large proportions of five- and six-times carboxylated benzene polycarboxylic acids (benzene pentacarboxylic acid = B5CA and mellitic acid = B6CA) [68], [69]. In this study, B6CA contributed up to 40% and 44% of total BC, respectively, in the pit fillings and recent topsoils (Table 6). Such large B6CA proportions (>40% B6CA of total BC) are typical for high burning temperatures in charcoals (i.e. 600 °C) [44], [45]. In the recent topsoils, the B6CA enrichment is most likely a result of modern fossil fuel combustion [117]. Yet, also the large contribution of B6CA to total BC in the pit fillings indicates human-induced fires on ancient arable fields, as these kinds of fires usually burn at higher temperatures compared with natural grass and forest fires ([45]; 23–33% B6CA of total BC in natural and forest fires). Indeed, in our study both, the subsoil samples and the humic zone, revealed elevated proportions of B4CA but only small proportions of B6CA to total BC (averagely 28% and 30%, respectively; Table 6) likely representing the “low temperature” natural fire background.

Summarizing, all studied arable topsoil relicts (pit filling material) were characterized by elevated amounts of fire-derived carbon, pointing to a human use of fire on prehistoric arable land. These fires were controlled burning events, which are also indicated by a change in BC quality. The early farmers might have used fire to clear the fields from understorey vegetation after cutting the trees [127]. They could have been aware of the benefits of periodically burning biomass on arable fields, i.e. albedo effect, weed suppression, liming, soil amelioration and fertilization [41], [42], [128]. However, in the studied regions with neutral to alkaline soil reaction, the liming effect was less important than the fertilizer effect due to the addition of plant available nutrients like calcium, magnesium, potassium and phosphorus from the burned biomass (mainly from the ash) [108], [109], [129] and the amelioration effects on physical, chemical, and biological soil properties [70], [130].

## Conclusions

We used a multi-proxy approach for studying the (micro-) nutrient status and the organic matter of Neolithic and Bronze Age arable topsoil relicts. The results showed:

- i.) that topsoil relicts did not hint at any nutrient deficiencies in Neolithic and Bronze Age arable soils, similar to findings for prehistoric phosphorus [8].
- ii.) that it was not possible to identify legumes by amino acid measurements; yet, D/L amino acid patterns pointed to the preservation of aged and microbially altered N forms in the prehistoric pit fillings.
- iii.) an enrichment of heavy ^15^N isotope that give first indications of prehistoric manuring.
- iv.) manure- (faeces-) derived C in the pit fillings that was verified by the detection of stable bile acids. They proved to be better indicators of prehistoric faeces additions than coprostanol and 5β-stigmastanol.
- v.) that arable fields had received combustion residues (black carbon), most likely derived from human-induced biomass burning.

Overall, the studied soils were fertile in prehistory. On the one hand, this reflects the favorable characteristics of the fertile loess soils as well as the low prehistoric population density. On the other hand, there were several hints that the fertility of the soils was sustained or even enhanced in prehistory, notably by additions of organic materials like charcoal and manure (faeces). Whether this was done intentionally remains unsure.

## Supporting Information

Figure S1 **Flow chart of steroid analyses according to Birk** ***et al.*** **** [49] **.**
(TIF)

Figure S2 **Products of sterol reduction to 5β-stanols in the gut of mammals, the subsequent conversion to epi-5β-stanols under anaerobic conditions, and the reduction products of sterols in soils (i.e. 5α-stanols and only to a minor extent 5β-stanols) (modified after Bull** ***et al.*** **** [30] **and Birk** ***et al.*** **** [131] **).** * If an input of algae can be excluded these steroids are animal-derived. The sources of the other unmarked steroids are e.g. plant litter, root exudates, and faeces (of herbivores and omnivores).
(TIF)

Table S1 **Contents of** ***aqua regia*** **extractable elements (in mg kg^−1^) of all pit filling soil samples.**
(PDF)

Table S2 **Molecular structures, the retention times, and the selected characteristic ion fragments of the relevant steroids.**
(PDF)

Table S3 **Bile acid contents (in µg kg^−1^ soil) (average and standard deviation in parentheses) in all investigated samples.**
(PDF)

Table S4 BPCA analysis (in duplicate) of all soil samples (average ± standard deviation; SD<15% for BPCAs; n.d., not detectable) and L* value.
(PDF)
