# Supplementary figures and images for: Organic Fertilization and Sufficient Nutrient Status in Prehistoric Agriculture? – Indications from Multi-Proxy Analyses of Archaeological Topsoil Relicts
Source: PLoS One. 2014 Sep 2;9(9):e106244. doi: 10.1371/journal.pone.0106244 (PMC4152168; doi:10.1371/journal.pone.0106244)

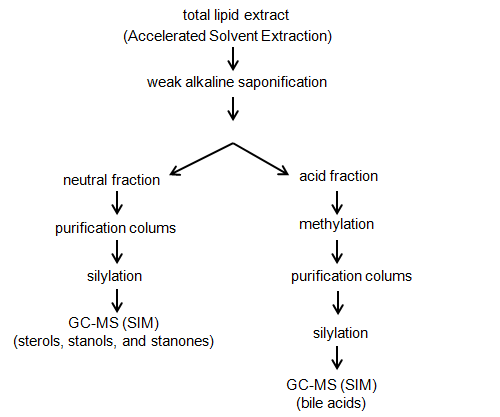

Supplement: Figure S1 — Flow chart of steroid analyses according to Birk et al. [49] . (TIF) [file pone.0106244.s001.tif]
