# Supplementary material for: Organic Fertilization and Sufficient Nutrient Status in Prehistoric Agriculture? – Indications from Multi-Proxy Analyses of Archaeological Topsoil Relicts
Source: PLoS One. 2014 Sep 2;9(9):e106244. doi: 10.1371/journal.pone.0106244 (PMC4152168; doi:10.1371/journal.pone.0106244)
Supplement: Table S1 — Contents of aqua regia extractable elements (in mg kg−1) of all pit filling soil samples. (PDF) [file pone.0106244.s003.pdf]

Table S1: Contents of *aqua regia* extractable elements (in mg kg^{-1}) of all pit filling soil samples.

Id	Site	Sample type	Ca	Co	Cu	Fe	I	Mg	Mn	Mo	Ni	P	S	Se	Sr	Ti	Zn	Zr
			[mg kg^{-1}]															
1	Klein Gräfendorf	pit filling	14819	6	5	18762	0	7392	410	0	15	348	788	0	52	421	37	10
2	Klein Gräfendorf	adjacent subsoil	14878	5	3	12925	0	7701	246	0	12	337	333	0	38	414	21	9
3	Klein Gräfendorf	recent topsoil	9796	7	16	21306	216	3974	490	1	18	683	361	9	34	445	44	8
4	Klein Gräfendorf	pit filling	57077	6	13	18568	151	8723	388	2	15	428	3189	8	53	424	25	12
5	Klein Gräfendorf	adjacent subsoil	81830	6	9	16973	150	11840	318	2	15	416	1435	7	59	408	19	17
6	Jüdendorf	adjacent subsoil	67953	6	4	17075	52	10809	337	0	15	385	95	0	56	441	31	8
7	Jüdendorf	pit filling	17676	7	4	19431	64	5602	464	0	17	378	675	0	24	429	40	6
8	Jüdendorf	recent topsoil	13244	7	7	23121	0	4736	441	0	18	463	240	0	31	585	50	19
9	Jüdendorf	pit filling	41488	7	26	19252	156	7255	375	2	17	402	796	9	29	442	30	18
10	Jüdendorf	adjacent subsoil	22905	7	15	21973	83	6392	492	0	17	371	1160	0	26	598	49	12
11	Jüdendorf	recent topsoil	4974	8	19	23380	69	3924	511	0	20	389	567	0	27	573	56	11
12	Jüdendorf	pit filling	12746	8	17	21709	69	5002	519	0	19	282	1083	0	18	578	47	8
13	Jüdendorf	adjacent subsoil	59553	7	14	19096	60	11372	363	0	15	401	737	0	53	548	36	10
14	Jüdendorf	recent topsoil	5126	8	25	21259	61	3382	584	1	18	417	702	0	29	473	67	8
15	Oechlitz	settlement pit	14709	6	7	16946	0	5896	416	0	15	2365	190	0	80	366	39	3
16	Oechlitz	pit filling	37994	6	12	17986	667	7950	421	2	16	873	1001	8	56	421	37	23
17	Oechlitz	adjacent subsoil	48628	7	9	18489	398	12025	316	2	15	843	754	8	60	473	27	52
18	Oechlitz	recent topsoil	11131	7	15	20077	645	4423	476	3	19	1339	179	10	35	466	42	31
19	Prießnitz	recent topsoil	3062	9	13	25641	270	4724	652	1	16	587	224	10	18	621	43	3
20	Prießnitz	pit filling	3895	9	13	27630	320	5935	1207	1	31	674	16	10	11	475	7299	16
21	Prießnitz	adjacent subsoil	31639	10	13	27463	190	10772	1972	2	27	427	80	11	31	450	47	11
22	Prießnitz	pit filling	5163	9	13	25143	138	6569	511	1	22	513	35	11	19	598	54	10
23	Prießnitz	adjacent subsoil	36970	9	16	25268	76	7257	468	1	22	322	43	9	34	366	35	11
24	Merzenich	recent topsoil	5930	8	15	23397	12	3857	652	3	21	1225	243	7	11	337	66	2
25	Merzenich	adjacent subsoil	54463	9	12	22880	224	7513	511	3	23	701	65	9	56	454	39	10
26	Merzenich	pit filling	4398	11	18	38374	424	4840	677	2	29	938	58	11	14	442	62	8
27	Merzenich	pit filling	3709	11	15	32349	372	4737	624	1	28	833	35	10	12	408	52	7
28	Merzenich	pit filling	4107	10	16	32326	348	4187	572	2	26	778	39	10	13	479	54	12
29	Merzenich	pit filling	4911	13	19	25494	519	4958	925	1	27	950	80	17	13	434	108	21
30	Merzenich	pit filling	3908	13	14	25031	282	3756	841	2	25	733	86	16	11	400	90	16
31	Merzenich	adjacent subsoil	25853	11	15	30412	6	3495	783	3	28	523	40	8	35	265	41	13
32	Merzenich	adjacent subsoil	2186	13	16	30632	4	2160	811	3	30	550	25	8	10	233	48	13
33	Merzenich	adjacent subsoil	2175	13	17	34812	7	2175	906	3	32	604	24	9	10	249	49	14
34	Merzenich	adjacent subsoil	3545	9	14	30942	18	3502	568	3	22	787	24	10	13	451	46	7
35	Merzenich	adjacent subsoil	4674	5	13	30729	21	5243	422	4	24	874	48	11	12	699	49	9
36	Merzenich	pit filling	3345	13	18	23914	423	3715	1017	1	23	661	61	14	13	514	61	14
37	Merzenich	pit filling	3168	15	21	26766	318	4281	952	2	29	727	87	18	15	40	75	14
38	Merzenich	adjacent subsoil	2398	18	26	29901	292	3721	1559	2	32	593	56	15	12	285	73	16
39	Merzenich	pit filling	4705	9	19	31488	12	4576	602	3	25	978	40	10	13	453	54	6
40	Merzenich	adjacent subsoil	5135	10	17	35850	15	6204	586	4	30	1269	43	10	12	500	50	7
41	Merzenich	pit filling	1034	9	14	48377	2	2508	672	2	20	552	21	5	6	86	28	4
42	Merzenich	adjacent subsoil	21537	18	23	40573	170	5334	1741	2	37	559	52	9	26	184	47	16
43	Merzenich	pit filling	5410	12	18	31154	404	5198	806	2	33	883	52	11	15	439	38	6
44	Merzenich	adjacent subsoil	50378	9	15	28181	10	8310	627	3	23	507	72	7	48	236	40	11
45	Pulheim	recent topsoil	2426	7	11	18430	207	2484	627	2	18	800	137	7	10	375	52	1
46	Pulheim	adjacent subsoil	3329	10	24	29483	0	4305	524	0	29	725	48	0	13	452	80	6
47	Pulheim	pit filling	3200	9	14	28203	236	4387	427	3	26	817	29	11	9	465	43	8
48	Pulheim	adjacent subsoil	2527	9	12	24576	194	3993	508	3	25	740	42	10	10	422	40	5
49	Pulheim	pit filling	4080	10	27	32206	0	4784	583	0	31	856	67	0	15	452	61	6
50	Düren Arnoldsweiler	below humic zone	1463	6	7	15526	86	2032	353	1	19	203	72	7	8	434	32	17
51	Düren Arnoldsweiler	humic zone	2070	9	13	19484	109	2399	767	1	30	257	62	10	10	430	39	15
