# Supplementary material for: Organic Fertilization and Sufficient Nutrient Status in Prehistoric Agriculture? – Indications from Multi-Proxy Analyses of Archaeological Topsoil Relicts
Source: PLoS One. 2014 Sep 2;9(9):e106244. doi: 10.1371/journal.pone.0106244 (PMC4152168; doi:10.1371/journal.pone.0106244)
Supplement: Table S2 — Molecular structures, the retention times, and the selected characteristic ion fragments of the relevant steroids. (PDF) [file pone.0106244.s004.pdf]

Table S2: Molecular structures, the retention times, and the selected characteristic ion fragments of the relevant steroids.

Biomarker group	Substance	Trivial name, molecular formular	Structure	Retention time (min)[a]	Characteristic ion fragments (m/z)[a]
**Sterols**	Cholest-5-en-3β-ol	Cholesterol<br>$C_{27}H_{46}O$	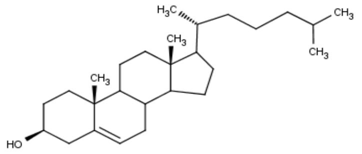	36.3	329; 368; 458
	Stigmast-5-en-3β-ol,<br>24-Ethyl-5-cholesten-3β-ol	β-Sitosterol<br>$C_{29}H_{50}O$	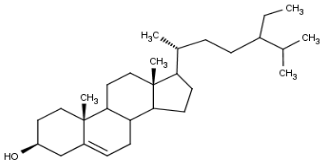	41.4	357; 396; 486
	Stigmasta-5,22-dien-3β-ol	Stigmasterol<br>$C_{29}H_{48}O$	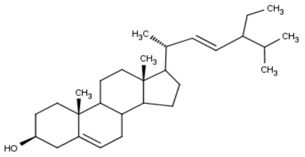	43.6	255; 394; 484
	5β-pregnan-3α-ol	Desoxypregnanolone[b]<br>$C_{21}H_{35}O$	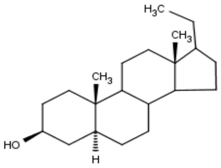	21.2	215; 286; 361
**Stanols**	5β-cholestan-3β-ol	**Coprostanol**<br>$C_{27}H_{48}O$	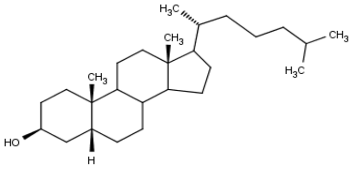	33.3	215; 257; 370
	5β-cholestan-3α-ol	**Epicoprostanol**<br>$C_{27}H_{48}O$	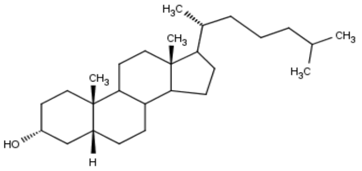	33.5	215; 355; 370
	5α-Cholestanol	**α-Cholestanol**<br>(Dihydrocholesterol)<br>$C_{27}H_{48}O$	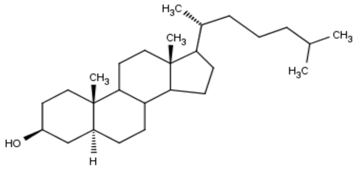	36.7	217; 445; 460
	24-ethyl-5β-cholestan-3β-ol	**β-Stigmastanol**<br>(24-Ethyl-Coprostanol)<br>$C_{29}H_{52}O$	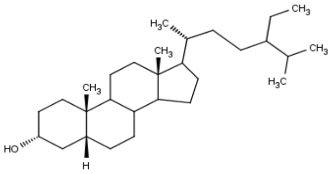	40.5	215; 383; 398
	24β-ethyl-5β -cholestan-3α-ol	**Epi-stigmastanol**<br>$C_{29}H_{52}O$	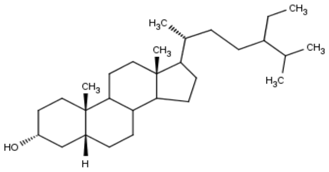	40.5	215; 383; 398
	24α-Ethyl-5α-cholestan-3β-ol	**α-Stigmastanol**<br>$C_{29}H_{52}O$	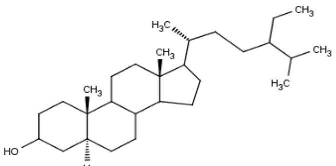	44.1	215; 383; 398

**Stanones**	5α-Pregnan-3-one	**Pregnanolone**[b,c]<br>$C_{21}H_{34}O$	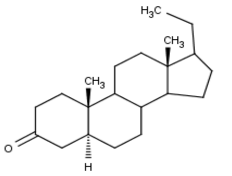	24.6	215; 300; 375
	5α-Cholestan-3-one	**Cholestanone**<br>$C_{27}H_{46}O$	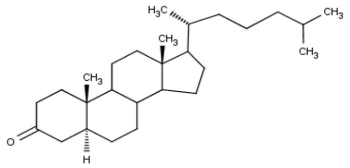	35.0	231; 386
	5β-Cholestan-3-one	**Coprostanone**<br>$C_{27}H_{46}O$	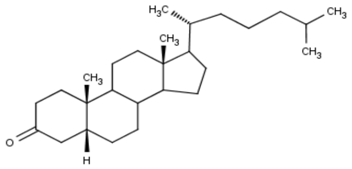	36.5	231; 386
	4-Cholesten-3-one	**4-Cholesten-3-one**<br>$C_{27}H_{44}O$	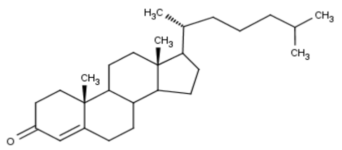	39.3	229; 261; 384
	5α-Cholestan-3β-ol-6-one	**6-Ketocholestanol**<br>$C_{27}H_{46}O_2$	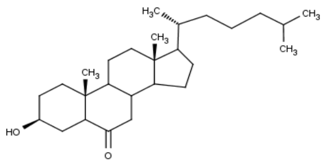	46.6	445; 459; 474
**Bile acids**	7α,12α-Dihydroxy-5β-cholanoic acid	Isodeoxycholic acid[d] (IDCA)<br>$C_{24}H_{40}O_4$	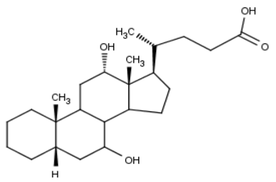	31.6	255; 355; 370
	3β-Hydroxy-5β-cholanoic acid	Isolithocholic acid (ILCA)<br>$C_{24}H_{40}O_3$	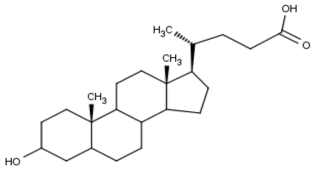	34.1	215; 257; 372
	3α-Hydroxy-5β-cholanoic acid	Lithocholic acid (LCA)<br>$C_{24}H_{40}O_3$	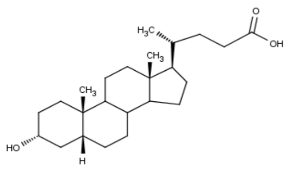	34.4	215; 257; 372
	3α,12α-Dihydroxy-5β-cholanoic acid	Deoxycholic acid (DCA)<br>$C_{24}H_{40}O_4$	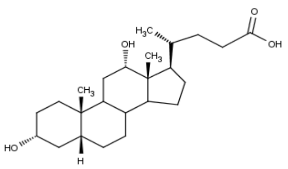	36.5	255; 345; 370
	3α,7α-Dihydroxy-5β-cholanoic acid	Chenodoxycholic acid (CDCA)<br>$C_{24}H_{40}O_4$	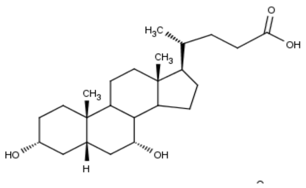	37.2	255; 355; 370

	3α, 6α -Dihydroxy-5β-cholanoic acid	Hyodeoxycholic acid (HDCA) $C_{24}H_{40}O_{4}$	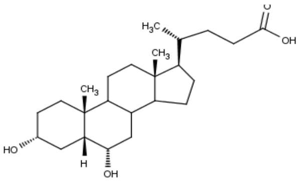	37.7	255; 355; 370
	3α, 7β -Dihydroxy-5β-cholanoic acid	Ursodeoxycholic acid (UDCA) $C_{24}H_{4}0O_{4}$	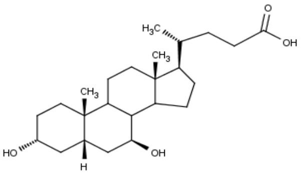	38.5	255; 355; 460
**second internal standard (IS2)**		5α-Cholestane[e]	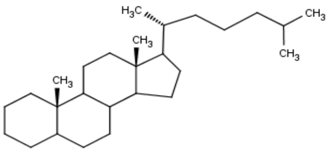	27.9[f]; 25.4[g]	271; 357; 372

[a] according to own measurements; [b] first internal standard for stanols and sterols; [c] first internal standard for stanones; [d] first internal standard for bile acids; [e] second internal standard; [f] measurement of stanols, stanones, and sterols; [g] measurement of bile acids.
