# Supplementary material for: Organic Fertilization and Sufficient Nutrient Status in Prehistoric Agriculture? – Indications from Multi-Proxy Analyses of Archaeological Topsoil Relicts
Source: PLoS One. 2014 Sep 2;9(9):e106244. doi: 10.1371/journal.pone.0106244 (PMC4152168; doi:10.1371/journal.pone.0106244)
Supplement: Table S3 — Bile acid contents (in µg kg−1 soil) (average and standard deviation in parentheses) in all investigated samples. (PDF) [file pone.0106244.s005.pdf]

Table S3: Bile acid contents (in µg kg^{-1} soil) (average and standard deviation in parentheses) in all investigated samples.

Region/ excavation site	sample type	soil depth [cm]	bile acids [µg kg^{-1} soil] Lithocholic acid	Deoxycholic acid	Chenodeoxy-cholic acid	Hyodeoxy-cholic acid	Ursodeoxy-cholic acid	Σ bile acids
**Western Germany**								
Luvisol region								
Merzenich	pit filling 1	123	16.7	536.5	22.4	134.0	31.9	741.5
	abjacent subsoil 1	123	n.d.	43.4 (2.8)	n.q.	28.9	18.1	90.4
	pit filling 2	125	n.q.	83.2 (38.6)	n.q.	59.7 (33.1)	n.q.	142.9
	abjacent subsoil 2	125	n.q.	53.4 (51.7)	n.q.	33.8 (47.8)	n.q.	87.2
	pit filling 3	130	n.d.	107.5 (0.9)	n.q.	170.4 (124.3)	n.d.	277.9
	adjacent subsoil 3	130	n.q.	n.q.	n.q.	31.8 (9.1)	n.d.	31.8
	pit filling 4	175	n.q.	104.6 (12.7)	n.q.	79.2 (14.9)	13.4 (4.5)	197.2
	adjacent subsoil 4	175	n.q.	161.4 (102)	20.4 (11.5)	84.8 (52.0)	56.3 (40.9)	322.9
	pit filling 5	180	n.q.	76.1 (46.6)	n.q.	73.9 (53.9)	n.q.	149.9
	adjacent subsoil 5	180	n.q.	21.6 (5.3)	n.q.	14.7 (4.6)	n.q.	36.3
	pit filling 6	263	16.8 (1.0)	214.0 (58.0)	n.q.	175.7 (56.3)	n.q.	406.5
	adjacent subsoil 6	263	n.q.	n.q.	n.q.	n.d.	n.q.	0.0
Düren Arnoldsweiler	humic zone	170	n.d.	n.d.	n.q.	n.d.	n.q.	0.0

n.q. not quantifiable (under quantification limit; 5 µg kg^{-1} soil for deoxcholic acid and 10 µg kg^{-1} soil for all other bile acids);

n.d. not detectable (under detection limit)
