# Supplementary material for: Organic Fertilization and Sufficient Nutrient Status in Prehistoric Agriculture? – Indications from Multi-Proxy Analyses of Archaeological Topsoil Relicts
Source: PLoS One. 2014 Sep 2;9(9):e106244. doi: 10.1371/journal.pone.0106244 (PMC4152168; doi:10.1371/journal.pone.0106244)
Supplement: Table S4 — BPCA analysis (in duplicate) of all soil samples (average ± standard deviation; SD<15% for BPCAs; n.d., not detectable) and L* value. (PDF) [file pone.0106244.s006.pdf]

Table S4: BPCA analysis (in duplicate) of all soil samples (average ± standard deviation; SD < 15% for BPCAs; n.d., not detectable) and L* value.

Id	Site	Sample type	BPCA-C [g kg^{-1} soil]	Σ B3CA-C [g kg^{-1} soil]	Σ B4CA-C [g kg^{-1} soil]	Σ B5CA-C [g kg^{-1} soil]	Σ B6CA-C [g kg^{-1} soil]	Colour [L*]
1	Kleingräfendorf	pit filling	118.1 ± 4.9	3.8 ± 0.1	22.6 ± 1.0	40.6 ± 1.6	51.1 ± 2.1	50.0
2	Kleingräfendorf	adjacent subsoil	46.5 ± 4.1	1.3 ± 0.1	20.4 ± 2.2	11.5 ± 0.8	13.3 ± 1.1	64.2
3	Kleingräfendorf	recent topsoil	60.9 ± 1.0	2.4 ± 0.1	12.6 ± 0.2	20.1 ± 0.2	25.8 ± 0.5	45.2
4	Kleingräfendorf	pit filling	124.6 ± 10.3	3.4 ± 0.2	22.7 ± 1.3	48.9 ± 3.8	49.7 ± 5.0	53.4
5	Kleingräfendorf	adjacent subsoil	24.3 ± 1.2	0.8 ± 0.0	11.5 ± 0.3	5.8 ± 0.4	6.2 ± 0.5	63.0
6	Jüdendorf	adjacent subsoil	30.4 ± 2.5	1.2 ± 0.1	14.0 ± 1.2	6.6 ± 0.5	8.5 ± 0.7	64.5
7	Jüdendorf	pit filling	110.3 ± 12.7	5.3 ± 0.8	22.9 ± 2.5	37.9 ± 4.9	44.1 ± 4.5	50.1
8	Jüdendorf	recent topsoil	32.9 ± 3.5	1.6 ± 0.2	9.9 ± 0.5	11.2 ± 0.5	10.2 ± 4.7	53.5
9	Jüdendorf	pit filling	68.9 ± 0.2	2.4 ± 0.0	15.6 ± 0.1	23.3 ± 0.1	27.6 ± 0.1	56.8
10	Jüdendorf	adjacent subsoil	104.7 ± 0.9	3.9 ± 0.0	22.1 ± 0.2	36.2 ± 0.4	42.5 ± 0.3	48.5
11	Jüdendorf	recent topsoil	77.3 ± 0.1	3.0 ± 0.1	15.7 ± 0.1	26.2 ± 0.0	32.4 ± 0.1	48.8
12	Jüdendorf	pit filling	114.9 ± 10.9	4.0 ± 1.2	22.6 ± 0.1	42.5 ± 8.1	45.7 ± 3.8	46.4
13	Jüdendorf	adjacent subsoil	n.d.	n.d.	n.d.	n.d.	n.d.	63.5
14	Jüdendorf	recent topsoil	77.0 ± 3.9	2.2 ± 0.1	14.9 ± 0.4	29.9 ± 1.4	30.0 ± 2.0	46.8
15	Oechlitz	settlement pit	68.6 ± 3.5	2.7 ± 0.1	13.8 ± 0.6	23.5 ± 1.3	28.6 ± 1.5	53.1
16	Oechlitz	pit filling	109.9 ± 1.7	3.3 ± 0.2	22.2 ± 1.1	44.3 ± 0.8	40.1 ± 0.4	52.9
17	Oechlitz	adjacent subsoil	29.7 ± 1.4	2.2 ± 0.2	13.6 ± 0.1	7.6 ± 0.7	6.3 ± 0.4	61.9
18	Oechlitz	recent topsoil	111.2 ± 2.0	3.9 ± 0.0	22.4 ± 0.0	38.6 ± 0.8	46.3 ± 1.2	46.4
19	Prießnitz	recent topsoil	33.9 ± 1.3	1.2 ± 0.1	8.4 ± 0.2	11.2 ± 0.6	13.2 ± 0.5	57.6
20	Prießnitz	pit filling	100.5 ± 23.2	4.9 ± 0.1	26.5 ± 0.1	36.9 ± 2.1	32.3 ± 21.1	55.2
21	Prießnitz	adjacent subsoil	55.9 ± 0.0	2.8 ± 0.0	19.7 ± 0.0	21.3 ± 0.0	12.1 ± 0.0	64.4
22	Prießnitz	pit filling	138.4 ± 12.0	5.9 ± 0.7	30.5 ± 3.1	48.0 ± 6.7	54.1 ± 1.5	50.4
23	Prießnitz	adjacent subsoil	10.2 ± 0.2	0.6 ± 0.0	5.0 ± 0.1	2.2 ± 0.0	2.5 ± 0.1	63.8
24	Merzenich	recent topsoil	37.5 ± 0.2	1.2 ± 0.0	7.4 ± 0.0	13.4 ± 0.1	15.5 ± 0.1	54.1
25	Merzenich	adjacent subsoil	25.8 ± 2.6	1.3 ± 0.4	12.4 ± 1.9	6.0 ± 0.2	6.1 ± 0.2	61.2
26	Merzenich	pit filling	182.3 ± 0.7	7.3 ± 0.0	42.7 ± 0.1	69.1 ± 0.3	63.2 ± 0.3	51.1
27	Merzenich	pit filling	153.8 ± 16.6	7.3 ± 1.1	51.4 ± 6.4	46.0 ± 4.8	49.2 ± 4.3	56.6
28	Merzenich	pit filling	148.0 ± 57.9	11.1 ± 0.8	44.5 ± 4.0	53.7 ± 16.4	38.7 ± 36.6	53.3
29	Merzenich	pit filling	141.3 ± 3.4	7.0 ± 0.2	33.5 ± 0.9	49.0 ± 1.2	51.8 ± 1.2	52.0
30	Merzenich	pit filling	127.4 ± 5.6	5.8 ± 0.3	28.7 ± 1.0	43.3 ± 2.2	49.7 ± 2.1	51.7
31	Merzenich	adjacent subsoil	10.1 ± 0.1	0.2 ± 0.3	4.8 ± 0.1	2.5 ± 0.0	2.6 ± 0.1	58.2
32	Merzenich	adjacent subsoil	n.d.	n.d.	n.d.	n.d.	n.d.	56.9
33	Merzenich	adjacent subsoil	n.d.	n.d.	n.d.	n.d.	n.d.	57.0
34	Merzenich	adjacent subsoil	96.5 ± 1.5	3.0 ± 0.1	31.5 ± 1.1	32.9 ± 0.8	29.1 ± 0.5	56.8
35	Merzenich	adjacent subsoil	88.9 ± 0.7	2.7 ± 0.2	25.0 ± 2.2	32.3 ± 0.5	28.9 ± 2.3	56.4
36	Merzenich	pit filling	222.1 ± 8.8	10.2 ± 0.2	51.4 ± 1.9	89.4 ± 3.3	71.2 ± 3.5	54.1
37	Merzenich	pit filling	191.3 ± 13.4	6.5 ± 0.2	39.7 ± 2.2	74.3 ± 5.4	70.8 ± 5.6	53.6
38	Merzenich	adjacent subsoil	n.d.	n.d.	n.d.	n.d.	n.d.	54.1
39	Merzenich	pit filling	224.4 ± 10.9	9.8 ± 0.8	50.6 ± 3.3	89.9 ± 5.1	74.2 ± 1.7	53.2
40	Merzenich	adjacent subsoil	n.d.	n.d.	n.d.	n.d.	n.d.	57.1
41	Merzenich	adjacent subsoil	n.d.	n.d.	n.d.	n.d.	n.d.	47.0
42	Merzenich	pit filling	44.7 ± 4.3	1.7 ± 0.1	21.8 ± 2.3	9.7 ± 0.8	11.5 ± 1.0	57.9
43	Merzenich	pit filling	64.3 ± 0.4	2.4 ± 0.2	24.2 ± 0.5	17.6 ± 0.4	20.1 ± 0.3	56.4
44	Merzenich	adjacent subsoil	7.5 ± 0.3	0.2 ± 0.0	3.2 ± 0.2	1.8 ± 0.1	2.2 ± 0.1	61.1
45	Pulheim	recent topsoil	53.9 ± 0.3	2.1 ± 0.4	11.6 ± 0.1	15.2 ± 0.1	24.9 ± 0.3	56.4
46	Pulheim	adjacent subsoil	95.6 ± 0.8	2.8 ± 0.1	24.6 ± 0.6	37.2 ± 1.3	31.0 ± 2.7	57.0
47	Pulheim	pit filling	126.0 ± 3.0	4.8 ± 0.2	42.5 ± 3.2	35.3 ± 0.3	43.3 ± 0.1	56.9
48	Pulheim	adjacent subsoil	101.9 ± 0.2	2.8 ± 0.1	26.5 ± 0.2	37.5 ± 0.2	35.1 ± 0.4	56.8
49	Pulheim	pit filling	143.2 ± 8.7	5.2 ± 0.2	30.6 ± 0.5	57.2 ± 2.2	50.2 ± 7.2	52.4
50	Düren Arnoldsweiler	below humic zone	n.d.	n.d.	n.d.	n.d.	n.d.	68.9
51	Düren Arnoldsweiler	humic zone	100.1 ± 4.1	3.7 ± 0.1	37.0 ± 1.0	29.0 ± 1.0	30.4 ± 2.0	62.2
